# A review of our current understanding of parasite survival in silage and stored forages, with a focus on *Fasciola hepatica* metacercariae[Link]

**DOI:** 10.1111/gfs.12429

**Published:** 2019-05-07

**Authors:** Bethan C. John, David R. Davies, Diana J. L. Williams, Jane E. Hodgkinson

**Affiliations:** ^1^ Institute of Infection and Global Health University of Liverpool Liverpool UK; ^2^ Silage Solutions Ltd Ceredigion UK

**Keywords:** *Fasciola hepatica*, metacercariae, silage, parasite, forages, food security

## Abstract

*Fasciola hepatica*, the parasitic liver fluke, is a re‐emerging zoonotic infection and an important cause of morbidity and mortality in ruminant livestock worldwide. A significant animal welfare concern, fasciolosis also has a detrimental impact on food security, with the effects of sub‐clinical infection on growth rate and milk yield estimated to cost the UK cattle industry £40.4 million annually. It is understood that up to 50% of infective *F. hepatica* metacercariae may overwinter on pasture and remain viable to infect grazing livestock the following spring. However, the infection risk posed by feeding grass silage to livestock remains largely unknown as the majority of previous studies are outdated in both experimental design and analysis of ensiled metacercariae viability. Anecdotal reports of fasciolosis outbreaks in housed livestock have reignited interest in *F. hepatica* metacercariae survival during modern ensiling processes and more importantly if they retain viability within stored forages. Consequently, a comprehensive review of the available literature is required in order to identify knowledge gaps and highlight future research opportunities regarding the role of silage in the transmission of *F. hepatica*.

## 1 INTRODUCTION

*Fasciola hepatica*, commonly referred to as liver fluke, is a parasitic trematode associated with temperate climates. It primarily affects sheep and cattle but other species including horses, deer, buffalo and camelids can be infected. Human fasciolosis has been identified as a re‐emerging zoonosis and neglected tropical disease (World Health Organisation (WHO) (2007)). Fluke are found in the liver and bile ducts of their definitive hosts, causing acute and chronic disease characterized by haemorrhage, anaemia, impaired liver function and weight loss. With regard to ruminant livestock, the impact of sub‐clinical infection with *F. hepatica* on growth rate and milk yield is estimated to cost the UK cattle industry £40.4 million annually (Bennett & Ijpelaar, 2005). Additional economic costs include those related to use of veterinary diagnostics and drug treatment, cost of biosecurity measures and loss of value due to carcass condemnation. The increasing prevalence of livestock fasciolosis is of major concern; there is also an increase in disease incidence. This has been attributed to many factors such as climate change and emerging anthelmintic drug resistance—particularly to triclabendazole, a commonly used treatment. Expanding livestock movements and changing farm management practices such as irrigation and implementing environmental wetland schemes also play an important role (Beesley et al., 2017; McCann, Baylis, & Williams, 2010; Pritchard, Forbes, Williams, Salimi‐Bejestani, & Daniel, 2005).

The lifecycle of *F. hepatica* is complex, due to its dependence on key climatic factors and the need for an intermediate host, a freshwater mud snail, *Galba truncatula*. Adult parasites in the bile ducts produce eggs that are shed onto pasture in faeces. Egg embryonation occurs only when temperatures exceed 10°C (Ross & McKay, 1929) resulting in the development and hatching of miracidia into water. The highly motile miracidium locates and penetrates the intermediate snail host where clonal amplification occurs, resulting in multiple identical intramolluscan stages known as cercariae. These tadpole‐like stages migrate through snail tissues and are stimulated to be shed when temperatures reach between 10°C and 26°C (Kendall & McCullough, 1951). Cercariae are very active; they frequently change direction and eventually encyst on blades of grass, as metacercariae—the infectious stage. Following attachment to a substrate, the survival of metacercariae is dependent on moisture and moderate temperatures. They have been demonstrated to survive repeated freeze‐thaw action (Boray & Enigk, 1964). Metacercariae are ingested from pasture by grazing livestock and undergo excystment within the small intestine. Newly hatched juvenile fluke migrate through the abdominal cavity towards the liver where maturation, reproduction and egg laying occur, re‐initiating the parasitic life cycle.

Given that grass silage is sourced from pasture, it is important to consider practices that may influence the infectivity of pasture used for ensiling. How pasture is managed impacts on metacercarial burden but practices vary considerably among farms and ultimately rely on restricting direct access of grazing livestock to habitats known to favour *G. truncatula*. Management practices which may indirectly reduce pasture cercariae and metacercariae contamination include the application of copper sulphate and nitrogenous fertilizers (Brglez & Wikerhauser, 1968). Spreading manure poses a risk to contamination of pasture; however, heat treatment of liquid manure at 50°C for 4 hr prior to spreading reduces the viability of *F. hepatica* eggs and subsequent contamination of fertilized pastures (Moazeni, Ansari‐Lari, Masoodfar, Hosseinzadeh, & Mootabi Alavi, 2010). Aeration of slurry results in a greater rate of aerobic decomposition and increases temperatures within waste vessels. Aeration trials conducted in both summer and winter conditions demonstrated that *F. hepatica* eggs contaminating slurry experienced 100% mortality regardless of seasonal variances, with maximum temperatures of 56°C in summer and 50°C in winter (Persson, 1973).

Within Western Europe, approximately 10 million hectares of grassland is harvested for silage production annually (Wilkinson & Toivonen, 2003). Silage provision is important in regions with restricted growing seasons or where pasture grazing is not possible all year round. Ensiling is achieved through the anaerobic fermentation of naturally occurring bacteria on the cut forage which produce lactic acid. The primary objective of ensiling is to produce a safe and reliable source of forage with the highest possible nutritional value for livestock. Nevertheless, a number of livestock pathogens are known to be associated with silage intake, such as the facultative anaerobic bacteria *Listeria monocytogenes* which can proliferate rapidly during aerobic spoilage (Fenlon, 1986). Ensiling of grass contaminated with animal carcasses or harvesting fodder from fields contaminated with fowl faeces are major risk factors for *Clostridium botulinum* transmission. Botulinum toxins are known to persist in cattle faeces for up to eight weeks after ingestion of contaminated brewers grains (Notermans, Breukink, Wensing, & Wagenaar, 1978), and spreading of manure from infected livestock has been demonstrated to contaminate pastures and stored cut forage if lactic acid fermentation is delayed (Notermans, Dufrenne, & Oosterom, 1981). If aerobic spoilage occurs, an increase in forage pH provides conditions favourable for fungal proliferation and mycotoxin production. Pre‐harvest mycotoxins, such as fungal alkaloids, may also persist throughout fermentation, which may affect livestock production, for example, by instigating antimicrobial effects on rumen microflora and impairing nutrient absorption (Fink‐Gremmels, 2008).

Approximately 50% of the *F. hepatica* metacercariae on pasture are reported to successfully overwinter and remain viable to infect livestock the following spring (Ollerenshaw, 1971). However, there is little research on the survival of *F. hepatica* metacercariae in silage, with only historic records available in the literature. Silage production and forage management practices have evolved since the earliest literature was published, with the machinery and systems currently used on farms surpassing their earlier counterparts in both harvest efficiency and crop yield (Wilkinson, 2005). In addition, protocols for laboratory‐based ensiling and methods for determining viability of metacercariae post‐ensiling have improved, due to the use of small‐scale, replicable ensiling vessels (O’Kiely & Wilson, 1991) and refinement of in vitro excystment assays. Following advancements in the detection of *F. hepatica* DNA from low‐yielding lifecycle stages including metacercariae (Cwiklinski et al., 2015a), there is potential to detect parasite contamination within the environment. A growing body of evidence suggests that *F. hepatica* has the ability to modulate livestock immune responses (Clery, Torgerson, & Mulcahy, 1996; Dalton, Robinson, Mulcahy, O'Neill, & Donnelly, 2013) and has evolved to overcome anthelmintic drug actions (Kelley et al., 2016). *F. hepatica* has a high genetic diversity, high gene flow (Beesley, Williams, Paterson, & Hodgkinson, 2016) and large biotic potential. With a large genome (Cwiklinski et al., 2015b), novel *F. hepatica* strains may rapidly evolve with selection of genes resulting in resistance to environmental pressures, including temperatures and pH conditions associated with anaerobic fermentation, which may result in improved metacercariae survival within silage. In addition, historical *F. hepatica* strains used in previous ensiling research may not be representative of the population of metacercariae contaminating pastures in the present day. As such, historic conclusions and findings on the viability of livestock parasites within silage, in particular *F. hepatica* metacercariae, require re‐evaluation. The survival of *F. hepatica* metacercariae within unfermented forages, such as hay and straw, will be reviewed to provide a broader perspective of the role of forage‐based diets in fasciolosis transmission.

## 2 *FASCIOLA* SPP*.* METACERCARIAE SURVIVAL IN SILAGE

Previous investigations into *F. hepatica* metacercariae survival and viability in silage were conducted by inoculating viable metacercariae onto pre‐ensiled grass, rather than pre‐seeding them onto grass prior to ensiling and therefore exposing them to the complete anaerobic fermentation process. One study involved the recovery of metacercariae which had been inoculated onto ensiled forage and subsequent experimental infection of 30 healthy rabbits (Tarczyñski & Podkówka, 1964). Three months post‐infection, post‐mortem revealed no pathological signs indicative of fasciolosis following infection with metacercariae exposed to ensiled forage for 3 days. However, a single rabbit developed hepatic lesions following infection with metacercariae exposed to ensiled forage for 24 hr. Following 23 days within ensiled forage, metacercariae appeared morphologically normal but lacked movement. It was concluded that viability of metacercariae is drastically reduced during the first 24 hr of exposure to ensiled forage, potentially as a consequence of lactic acid production and proteolytic bacteria proliferation during the initial fermentation stages. A similar experiment (Wikerhauser & Brglez, 1961) observed changes in the colour and shape of *F. hepatica* metacercariae which were inoculated onto grass and ensiled. An attempt to excyst 1566 ensiled metacercariae in vitro using digestive media demonstrated complete loss of viability following 35 and 57 days ensiling. Furthermore, following experimental infection of 24 guinea pigs with between 42 and 56 metacercariae per infection which were ensiled for 35 days, serology and post‐mortem data did not indicate presence of disease. Such results should be treated with caution when understanding the risk posed by silage to livestock fasciolosis, because the use of rodent experimental infection models may not always represent the course of infection observed in cattle and sheep (Rajcevic, 1929). Temperature of stored silage has been demonstrated to impact on the duration of *F. hepatica* metacercariae survival. A series of laboratory ensiling experiments conducted at 20°C and 40°C demonstrated that metacercarial viability was lost at 12 and 6 days respectively (Enigk, Hildebrand, & Zimmer, 1964). Research into the viability of *F. gigantica* metacercariae within preserved fodder provides a broader understanding of the survival of *Fasciola* species. A study in Hawaii involved the inoculation of approximately 700 viable *F. gigantica* metacercariae onto grass which was ensiled in 50‐gallon metal drums for 3 months (Alicata, 1938). Following fermentation, 200 metacercariae were recovered and orally administered to two guinea pigs. Twenty‐five days post‐infection, neither guinea pig presented pathology consistent with fasciolosis. Limitations of this study include the small number of experimentally infected hosts and low recovery (28.5%) of ensiled metacercariae for use in experimental infections. A parallel experiment following a similar protocol evaluated if the addition of an acidic preservative (hydrochloric and sulphuric acid) onto grass prior to ensiling, impacted on *F. gigantica* metacercariae survival and viability during fermentation. Approximately 300 ensiled metacercariae were recovered following 3 months storage and orally administered to two guinea pigs, both of which lacked adult parasites or hepatic lesions 25 days post‐infection. This study demonstrates that ensiling contaminated grass for at least 3 months may reduce the viability of *F. gigantica* metacercariae. The impact of acidic preservative on the viability of ensiled metacercariae could not be determined as both experiments resulted in complete loss of metacercarial viability.

## 3 *FASCIOLA* SPP*.* METACERCARIAE SURVIVAL IN OTHER STORED FORAGES

Inconsistencies exist in historic studies investigating *F. hepatica* metacercariae survival in hay, which are potentially due to variation in grass harvesting method and storage conditions. Hay naturally contaminated with metacercariae was typically used, and as a result, the origin, age and exact number of metacercariae involved in these experiments were unknown. The first study to investigate the survival of metacercariae in hay demonstrated that contaminated hay, stored for 8 months, was a source of infection to cattle, sheep and rabbits (Marek, 1927). Partially dried hay contaminated with *F. hepatica* metacercariae was shown to produce low levels of livestock infection following storage for three weeks (Nöller & Schmid, 1929); however, following 6 months storage, viability of metacercariae was lost. A study conducted in Croatia suggested that hay contaminated with *F. hepatica* metacercariae and stored outside can remain infective to sheep for up to 17 months (Rajcevic, 1929). Comparatively, naturally infected hay harvested on the Ethiopian highlands and stored under shelter was shown to pose a risk of infection to sheep for only 2 months (Njau & Scholtens, 1991). An experiment involving approximately 10,000 viable laboratory cultivated metacercariae was reported (Enigk & Hildebrand, 1964). Two haystacks were prepared, one consisting of damp grass and a second that contained dried grass; metacercariae were inoculated into each stack and stored for between 50 and 200 days. *Fasciola* spp. infection was confirmed by post‐mortem in six experimentally infected mice. Between 50% and 60% of the *F. hepatica* metacercariae stored within dried hay for 50 days were viable. By 200 days storage, all metacercariae within dried hay appeared deformed and there was no evidence of infection in mice 21 days post‐infection. After 50 days storage in damp hay only 35%–52% of metacercariae were viable; interestingly after 180 days storage 10% of the recovered metacercariae remained viable. This suggests that poorly stored, old hay originating from wet pastures contaminated with metacercariae may be a potential source of *F. hepatica* infection (Enigk & Hildebrand, 1964).

Further work investigated the impact of hay drying methods on *F. hepatica* metacercariae survival and viability (Tarczyñski & Szepelski, 1970). Hay dried on tripods contained the greatest number of viable metacercariae, subsequently confirmed by experimental infection of rodents, leading to the conclusion that preservation of forage by drying on tripods is inadequate as a means of killing *F. hepatica* metacercariae. Approximately 75% of metacercariae in ventilator fan‐dried hay perished and disease incidence was reduced by 59%, suggesting that rigorous drying protocols can reduce the infection risk from contaminated hay. In support of these results, experiments maintaining metacercariae in climate chambers calibrated to specific temperature and relative humidity (RH) levels showed that metacercariae require moisture and moderate temperatures for survival. When maintained at 10°C and 90% RH, *F. hepatica* metacercariae remain viable for 4 months on hay; however, when temperatures increase to 20°C they only remain viable for 2 weeks (Boray & Enigk, 1964). Consequently, inadequately dried hay stored in cold temperatures, for example outside during winter months, could pose an infection risk to sheep and cattle.

Studies on the survival of *F. gigantica* metacercariae within rice straw fodder has provided an evidence‐base for feeding regimes for stall‐fed buffaloes in Nepal (Mahato & Harrison, 2005). The warmer temperatures and high humidity associated with the Nepalese climate is known to contribute to disease prevalence; *F. gigantica* metacercariae can survive up to 4 months on rice straw when it is stored above 60% RH (Kimura & Shimizu, 1978). Local farming practices can also promote parasite transmission. Within developing regions, untreated livestock manure is commonly used as fertilizer, raising the possibility that fluke eggs may be spread onto otherwise uncontaminated pastures. Furthermore, water‐logged rice paddy fields provide an ideal habitat for the snail intermediate host. *F. gigantica* metacercariae typically encyst on herbage at the water level; hence, the lower portion of the rice stalk is often highly contaminated (Suhardono, Roberts, & Copeman, 2006). Research indicates that buffaloes fed on a diet of rice straw with the lower 40cm of each blade removed do not succumb to infection. However, livestock fed the highly contaminated lower regions of rice straw present with gross pathological signs consistent with acute infection (Mahato & Harrison, 2005).

The effects of wastelage fermentation on *F. gigantica* metacercariae viability have been investigated by inoculating metacercariae into a wastelage premix comprising of buffalo faeces, molasses and rice straw (Gupta, Jakhmola, & Kamra, 1987). Following 20 days fermentation, metacercariae retrieved from the wastelage were orally administered to guinea pigs. Post‐mortems showed no evidence of *F. gigantica* infection suggesting that metacercariae were destroyed by the wastelage fermentation. Interpretation of these studies must be made with caution given that (a) rodents were used as definitive hosts, rather than the natural, ruminant host (b) samples sizes, number of metacercariae used and replicate power calculations were not stated in the literature.

## 4 NON‐*FASCIOLA* SPP*.* PARASITE SURVIVAL IN SILAGE AND OTHER STORED FORAGES

Paramphistomiasis, caused by the trematode *Calicophoron daubneyi,* is a livestock disease that is becoming increasingly prevalent in temperate climates (Huson, Oliver, & Robinson, 2017). Much like *F. hepatica*, *C. daubneyi* requires the intermediate snail host, *G. truncatula,* for life cycle completion. Co‐infection events have been documented in natural and laboratory‐maintained snail populations (Vignoles, Titi, Mekroud, Rondelaud, & Dreyfuss, 2017). *C. daubenyi* cercariae are shed onto pasture in a similar manner to those of *F. hepatica*; however being positively geotactic, they preferentially encyst under humid conditions associated with lower stem regions (Dreyfuss, Abrous, Vignoles, & Rondelaud, 2004). The question of *C. daubneyi* metacercariae survival in silage must also be raised; given that *F. hepatica* and *C. daubneyi* share similar niches, there is potential for *C. daubneyi* metacercariae to contaminate grass destined for silage production. To date, there are no studies investigating the persistence and survival of *C. daubneyi* metacercariae in silage or stored forages. As a result, the longevity of *C. daubneyi* metacercariae on pasture and their ability to overwinter, alongside their resilience to temperature, humidity and pH conditions, represents a substantial knowledge gap. Further research into cercariae encystment height on grass blades and how their geotactic nature potentially reduces the likelihood of silage contamination events due to encysting below the cut height is also required.

In terms of other parasites impacting on livestock health and welfare, ensiling grass contaminated with gastrointestinal nematode larvae such as *Ostertagia ostertagi* and *Cooperia oncophora* is thought to reduce the risk of infection, compared with permitting livestock to graze contaminated pastures. Following 50 days ensiling, larvae appear morphologically unviable provided that an acidic pH and anaerobic conditions are rapidly established; however, the addition of a formic acid additive was not found to accelerate larval destruction (Persson, 1974a). Third stage larvae of *Trichostrongylus colubriformis* and *Haemonchus contorus* retain viability when ensiled for 19 days at 20°C (Enigk et al., 1964), and viable nematode larvae have also been detected in hay after six weeks of drying (Persson, 1974b). The survival of strongyle eggs and larvae in wastelage and fermented liquid dung (FLD) has also been studied, with eggs inoculated into wastelage mixes and later examined microscopically for viability and development of larvae (Gupta, Singh, Jakhmola, & Kamra, 1985). At 15 days post‐fermentation, dead larvae were visible and no viable strongyle eggs were observed. By 20 days post‐fermentation, all eggs and larvae were destroyed, which is comparable with previous studies (Ciordia & Anthony, 1969). Factors contributing to the loss of viability and physical destruction of strongyle eggs and larvae were not ascertained; however, they were presumed to be associated with the acidic anaerobic environment and the presence of other microorganisms in the wastelage. While this study focusses on strongyle egg survival in wastelage, rather than trematode metacercariae, it does provide a deeper understanding of parasite survival following exposure to the fermentation processes involved in forage preservation.

It has been established that the unembryonated eggs of *Ascaris suum,* a nematode infection of pigs, and *Parascaris equorum*, a related parasite infecting horses, remain viable following ensiling and on recovery from contaminated forage continue to develop into adult worms (Pavlov, Tatarov, Lazarov, & Stoev, 1958). Experimental murine infections demonstrated that embryonated *A. suum* eggs lost viability following 5 months ensiling; however, embryo movement was detectable within embryonated eggs that were only ensiled for 2 months. The larval stages of the bovine lungworm *Dictyocaulus viviparus* appeared to lose motility and viability following only 30 days ensiling (Pavlov et al., 1958). The survival of cestode eggs from species such as *Echinococcus granulosus* and *Taenia multiceps* in maize and ryegrass silages also provide a useful insight into parasite survival in alternative forages. Eggs of *T. multiceps* lost viability following 15 days ensiling; however, *E. granulosus* oncospheres remained viable within silage for up to 45 days (Pavlov, Dimitrov, & Bratanov, 1969). It is apparent that survival rates of parasite infective stages in silage will depend on the parasite species, the source of material to be ensiled, the ensiling process and the length of time used for ensiling prior to feeding forage to livestock.

## 5 DISCUSSION

Silage production methods have advanced over the past 50 years; hence, the current risk posed by feeding silage and its impact on parasite transmission, specifically fasciolosis in livestock, remains unknown. Anecdotal reports of disease outbreaks in housed livestock have reignited an interest in understanding how long *F. hepatica* metacercariae survive during modern ensiling processes. This review recognizes that there are a number of knowledge gaps; for example, there is a need to identify specific factors during harvest, storage and fermentation that influence metacercarial survival and viability within silage. It is difficult to compare historical studies as they do not provide information on key fermentation processes or specify important factors such silage temperature, pH and organic acid composition (Enigk et al., 1964). There is a lack of information about the impact of a number of factors on metacercariae viability, such as grass Dry Matter (DM) content, the use of modern additives and inoculant products and, in particular, aerobic spoilage within the ensiling process.

The introduction of oxygen to ensiled forage through poor consolidation, inadequate sealing or poor feed‐out management is estimated to cost the UK agriculture sector £110 million annually (Williams, Critten, & Reynolds, 1995). Currently, there is lack of consideration given to the possibility of survival of *F. hepatica* metacercariae in silage when forage management and the resulting fermentation are poor. Research themes highlighted during the International Silage Conference 2018, for which proceedings are available (Gerlach & Südekum, 2018), included enhancing harvest efficiency, introducing novel AI technologies to increase crop production and development of improved diagnostic tools to detect livestock diseases, for example ketosis. However, it is vital that amidst innovation there is continued investigation into areas underpinning good silage production, such as adequate forage wilting and anaerobic sealing. Farmers are continually encouraged to follow standard management practices to economically produce a safe and highly nutritional product. Research into the impact of aerobic spoilage on *F. hepatica* metacercariae survival in silage, which reinforces the importance of basic forage management, should be considered of the utmost importance.

From a disease prevalence perspective, better understanding of trematode metacercarial survival rates, especially of highly pathogenic species such as *F. hepatica*, in silage produced using modern ensiling techniques, is needed. It would allow farmers to make informed decisions regarding on‐farm parasite control using an evidence‐based approach. At present, there are no commercialized livestock vaccines available which are effective against *F. hepatica* infection. A lack of vaccines places immediate pressure on the use of anthelmintic treatments to control *F. hepatica* infection; extensive resistance to flukicides has been documented and poses a risk to effective disease control. A greater understanding of survival of metacercariae within silage is vital when attempting to unravel the mechanisms involved in *F. hepatica* transmission and prevent excessive usage of anthelmintic treatments on farms.

## 6 CONCLUSION

This review has highlighted that there is a limited resource of publications describing the survival of *F. hepatica* metacercariae in stored forages; the majority of which are from several decades ago. Review of these papers has identified a number of knowledge gaps including inadequate sample size, exposing metacercariae to pre‐ensiled grass rather than pre‐inoculating grass prior to the fermentation process and failing to study specific fermentation conditions. Another limitation has been the use of non‐ruminant host species for experimental infections and relying on morphological analysis to determine metacercarial viability post‐ensiling. In addition, there is an absence of research on *C. daubneyi* metacercarial survival, which must be addressed given the re‐emergence of this species and potential for metacercarial contamination of harvested forage. Future opportunities to investigate the specific factors which impact on trematode metacercarial persistence and viability within silage and the risk posed by inadequate fermentation on parasite survival within stored forages should be prioritized.

## CONFLICT OF INTEREST

There are no conflicts of interest to declare.
